# Matrix Metalloproteinases in a Sea Urchin Ligament with Adaptable Mechanical Properties

**DOI:** 10.1371/journal.pone.0049016

**Published:** 2012-11-16

**Authors:** Ana R. Ribeiro, Alice Barbaglio, Maria J. Oliveira, Cristina C. Ribeiro, Iain C. Wilkie, Maria D. Candia Carnevali, Mário A. Barbosa

**Affiliations:** 1 INEB- Instituto de Engenharia Biomédica, NEWTherapies Group, Universidade do Porto, Porto, Portugal; 2 FEUP- Faculdade de Engenharia da Universidade do Porto, Departamento de Engenharia Metalúrgica e de Materiais, Porto, Portugal; 3 Life Sciences Department, University of Milan, Milano, Italy; 4 FMUP- Faculdade de Medicina da Universidade do Porto, Porto, Portugal; 5 ISEP-Instituto Superior de Engenharia do Porto, Departamento de Física, Porto, Portugal; 6 Department of Biological and Biomedical Sciences, Glasgow Caledonian University, Glasgow, Scotland; 7 ICBAS- Instituto de Ciências Biomédicas Abel Salazar, Universidade do Porto, Porto, Portugal; University of Minho, Portugal

## Abstract

Mutable collagenous tissues (MCTs) of echinoderms show reversible changes in tensile properties (mutability) that are initiated and modulated by the nervous system via the activities of cells known as juxtaligamental cells. The molecular mechanism underpinning this mechanical adaptability has still to be elucidated. Adaptable connective tissues are also present in mammals, most notably in the uterine cervix, in which changes in stiffness result partly from changes in the balance between matrix metalloproteinases (MMPs) and tissue inhibitors of metalloproteinases (TIMPs). There have been no attempts to assess the potential involvement of MMPs in the echinoderm mutability phenomenon, apart from studies dealing with a process whose relationship to the latter is uncertain. In this investigation we used the compass depressor ligaments (CDLs) of the sea-urchin *Paracentrotus lividus*. The effect of a synthetic MMP inhibitor - galardin - on the biomechanical properties of CDLs in different mechanical states (“standard”, “compliant” and “stiff”) was evaluated by dynamic mechanical analysis, and the presence of MMPs in normal and galardin-treated CDLs was determined semi-quantitatively by gelatin zymography. Galardin reversibly increased the stiffness and storage modulus of CDLs in all three states, although its effect was significantly lower in stiff than in standard or compliant CDLs. Gelatin zymography revealed a progressive increase in total gelatinolytic activity between the compliant, standard and stiff states, which was possibly due primarily to higher molecular weight components resulting from the inhibition and degradation of MMPs. Galardin caused no change in the gelatinolytic activity of stiff CDLs, a pronounced and statistically significant reduction in that of standard CDLs, and a pronounced, but not statistically significant, reduction in that of compliant CDLs. Our results provide evidence that MMPs may contribute to the variable tensility of the CDLs, in the light of which we provide an updated hypothesis for the regulatory mechanism controlling MCT mutability.

## Introduction

Echinoderms (starfish, sea urchins and others) have connective tissues with the unique ability to change mechanical properties such as elasticity and viscosity in short physiological time scales (<1 s to minutes) under nervous control [1], [2]. They are called either mutable collagenous tissues (MCTs) to reflect the reversibility of their tensile changes, or catch connective tissues due to their energy-sparing capacity to maintain body or appendage posture with low oxygen consumption [1]–[4]. Each shows one of three patterns of tensile change: (1) only reversible stiffening and destiffening (e.g. sea urchin peristomial membrane and compass depressor ligament) [5], [6]; (2) only irreversible destabilization, always associated with autotomy (defensive self-detachment) e.g. crinoid syzygial ligament [7]; (3) irreversible destabilization as well as reversible stiffening and destiffening (e.g. ophiuroid intervertebral ligament) [8]. MCTs are present in all five living echinoderm classes in several anatomical forms (dermal connective tissue, interossicular ligaments and tendons). Most MCT structures resemble mammalian connective tissues in that they consist largely of collagen fibril arrays, proteoglycans (PGs), fibrillin-containing microfibrils and water [2], [9]–[17].

MCTs are also characterized by the invariable presence of specialized neurosecretory-like cells known as juxtaligamental cells (JLCs) [2], [18]. There is evidence that the intracellular granules of JLCs store molecules that directly affect the interfibrillar cohesion of MCTs [2]. So far, only one such potential effector molecule has been identified and fully characterized. This is tensilin, a glycoprotein present in the dermis of holothurians (sea cucumbers) that forms interfibrillar bridges between collagen fibrils, preventing interfibrillar slippage and increasing the resistance of the tissue to tensile forces [19], [20]. Tensilin and another incompletely characterized molecule from holothurian dermis [21] may be regulatory stiffening agents. So far, no potential regulatory destiffening agents have been identified. It has been speculated that certain enzymes might have such a role. For example, since the C-terminus of tensilin, which contains a collagen-binding domain, is susceptible to proteolysis, it has been suggested that rapid destiffening of holothurian dermis could depend on the inactivation of tensilin by a specific protease [19]. The fact that the amino acid sequence of tensilin indicates 21–36% homology with mammalian tissue inhibitors of metalloproteinases (TIMPs) [19], raises the intriguing possibility that matrix metalloproteinases (MMPs) may be directly involved or that the regulatory mechanism has evolved from a MMP-TIMP system [2].

MMPs are a family of enzymes that can degrade all extracellular matrix (ECM) components and are extensively involved in the ECM remodelling that accompanies morphogenesis and wound healing in mammals [22]–[25], and development and regeneration in echinoderms [26]–[28]. Furthermore, MMPs contribute to the destiffening of the mammalian uterine cervix, which precedes and facilitates the dilatation of the cervix during fetal delivery [29]–[35]. Whilst the uterine cervix can also be regarded as a mutable collagenous structure, its changes in mechanical properties differ from those of echinoderm MCTs in having a much longer time course (hours to weeks) and in being under primarily endocrine rather than neural control [34]. Another important difference is that cervical destiffening is achieved partly through the degradation of collagen fibrils [30], [34], [35], whereas there is no evidence that this accompanies the destiffening of echinoderm MCTs and indeed the capacity of most of these tissues to rapidly restiffen makes this highly unlikely *a priori* [2], [36]. MMPs could, however, destabilize echinoderm MCTs by hydrolyzing non-collagenous components that contribute to interfibrillar cohesion. Such a mechanism may be responsible for the dermal liquefaction shown by some holothurians, which appears to result from the digestion of interfibrillar molecules (possibly proteoglycans) by a gelatinolytic enzyme that has no discernible effect on the collagen fibrils themselves [37].

Dermal liquefaction is an extreme (or even fatal) phenomenon whose relationship to reversible MCT mutability is unclear [37], [38]. We chose to explore the possible role of MMPs in the latter by using as a model a more conventional mutable collagenous structure – the compass depressor ligament (CDL) from the lantern (masticatory apparatus) of the sea urchin *Paracentrotus lividus* (Lam.). The sea urchin lantern contains ten CDLs, which, when stiff, help to stabilize the position of the lantern, and which destiffen to permit movement of the lantern by its intrinsic musculature [5]. We examined the effect of a broad-spectrum MMP inhibitor on the mechanical properties of CDLs in different tensile states and we used gelatin zymography to quantify MMPs in such CDLs. Our results provide evidence that MMPs may contribute to the variable tensility of the CDL, in the light of which we provide an updated hypothesis for the regulatory mechanism underpinning MCT mutability. It is proposed that in all mechanical states both activated MMPs and crosslink components are produced constitutively at a constant rate, and that the degree of interfibrillar crosslinking, and therefore stiffness, is regulated through changes in the rate of release of an endogenous MMP inhibitor.

## Materials and Methods

### Animal Tissues and Bathing Solutions

Adult individuals of *P. lividus* of similar size were collected in Aguda (north Portuguese coast) and maintained in an aquarium as described previously [36]. Isolated preparations of compass depressor ligaments (CDLs) were obtained from the lantern and mutability was mimicked as described previously [36]. In brief, the ‘compliant’ state was reproduced *in vitro* by immersing isolated CDLs for 45 minutes in 0.1% propylene phenoxetol (Sigma Aldrich 484423) in seawater (PPSW), which is an effective anaesthetic for echinoderms. The ‘stiff’ state was obtained by immersion of CDLs in 1 mM acetylcholine chloride (Sigma Aldrich 6625) in seawater (AChSW) for 15 min. Controls, which were in the ‘standard’ state, were kept in seawater (SW) alone. Although the CDL is partly delimited by a contractile myoepithelium, in *P. lividus* this occupies only around 8% of its total cross-sectional area, and we have shown previously that destiffening and stiffening due to PPSW and AChSW respectively result from changes in the passive mechanical properties of the collagenous component and not from effects on the myoepithelium (Wilkie, Fassini and Candia Carnevali, in preparation) [5].

### Mechanical Properties

#### Dynamic mechanical tests

**Figure 1:**
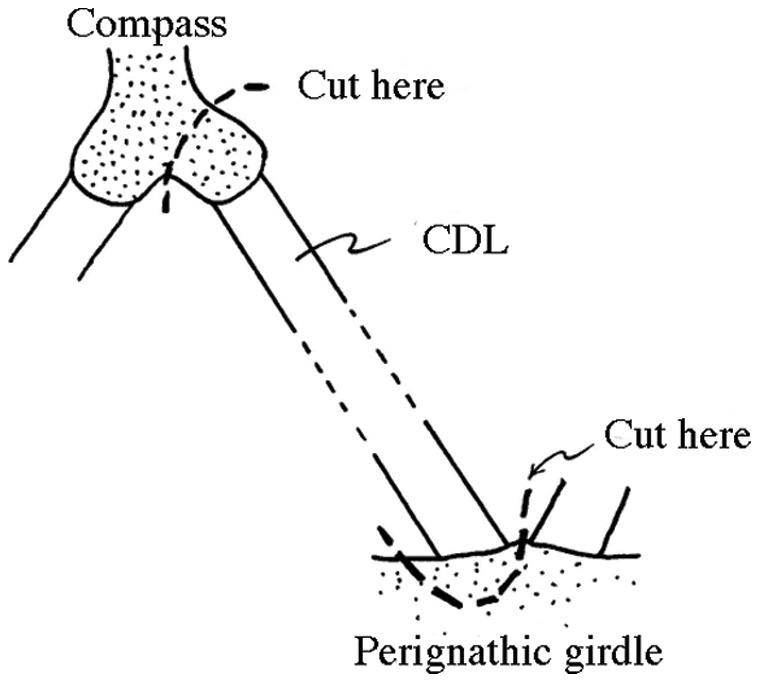
Schematic representation of the CDL dissection.

CDLs, which are strap-shaped bands of soft tissue 9–10 mm long, 0.2–0.4 mm wide and less than 0.1 mm thick, were dissected intact together with a small portion of the skeletal ossicles to which they were attached at both ends (Fig.1). The presence of the ossicle portions enabled the CDLs to be held firmly in the dynamic mechanical apparatus with minimal damage to the soft tissue. Fresh CDLs were always used and, before being analysed, were stored in SW. All CDLs were tested at a constant temperature of 20°C.

A dynamic mechanical analyser (DMA) (Tritec 2000; Triton Technology Ltd., Nottinghamshire, UK) with tension clamping geometry was used to determine the effect of chemical stimulation on the biomechanical properties of the CDL. Standard tension tests were chosen, because in the animal CDLs are continuously stretched due to routine movements of the lantern. The ossicles at the ends of each CDL were glued to the clamps of the equipment with cyanoacrylate glue, which was found to be suitable because of its rapid polymerization time (a few seconds). Beeswax (Sigma-Fluka 14367) was used as a coating, in order to avoid contact between the cyanoacrylate glue and testing solutions. The mean thickness of each CDL was determined by several measurements using a digital micrometer, and it was assumed that sample cross-section was circular (average diameter between 0.2–0.4 mm). After mechanical fixation, and before starting the mechanical tests, tissues were allowed to equilibrate for 10 min in seawater. During the experiments, sinusoidal force and displacement signals were measured simultaneously and this data set was resolved into complex modulus (E*), which is a measure of stiffness, and tan δ (damping), which is the ratio of the loss modulus (E′′) to the storage modulus (E). The storage modulus represents the elastic modulus in phase with the stress, while the loss modulus represents the viscous contribution to stiffness because it is an out-of-phase component. As the software provided E′, E′′ and tan δ, E* can be calculated by the following equation:
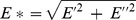



#### Viscoelasticity of CDLs in different mechanical states

CDLs were clamped and left in seawater for 10 min before being treated with propylene phenoxetol or acetylcholine, as described above, to reproduce the compliant and stiff states, respectively. Five animals, and at least three CDLs from each animal were used for each mechanical state. To determine the optimal experimental parameters, i.e. those that provided maximum differentiation between the three mechanical states, CDLs in all three conditions were subjected to two types of experiment: (1) In constant frequency experiments, the frequency was kept at 1 Hz and each CDL was subjected to a maximum strain which was changed stepwise in the following sequence: 5%, 10%, 15%, 10% and 5%, over the course of 5 sec (15% strain causes the rupture of some CDLs). (2) In constant strain tests, the maximum imposed strain was kept at 13% and each CDL was subjected to a frequency that changed stepwise as follows: 0.1 Hz, 0.3 Hz, 0.5 Hz, 0.7 Hz 1 Hz, 3 Hz, 5 Hz.

#### Effect of MMP inhibition on CDL viscoelasticity

The effect of the MMP inhibitor galardin (Calbiochem 364205) at 25 and 50 µM was investigated in CDLs using dynamic mechanical tests at 1 Hz and 13% maximum strain (the conditions that provide the maximum differentiation between the different mechanical states). Untreated CDLs were tested for 4 min before galardin was added. The duration of galardin inhibition was quantified as the time period between the point at which the E* maximized after addition of 50 µM galardin, and the point at which the E* returned to the value observed immediately before galardin addition. The effect of galardin on E* was quantified by normalizing the E* value when it started to decrease against the value just before the application of the chemical. The reversibility of the galardin effect was tested by immersing CDLs in the three mechanical states in SW, AChSW or PPSW containing 50 µM galardin, then rinsing the CDLs with SW, AChSW or PPSW alone and treating them again with 50 µM galardin in the respective solutions.

### Enzymatic Activity

#### Gelatinolytic activity in CDLs

Gelatinolytic activity was detected in more than five animals for each of the three mechanical states. Animals, from which the top half of the test (skeleton) had been removed to expose the intact lantern, were incubated in SW, AChSW and PPSW to obtain ligaments in the standard, stiff and compliant states, respectively. The CDLs were then quickly excised and placed in ice-cold RIPA lysis buffer (200 mMTris-HCl buffer pH 7.5 in 1% Triton-X 100, 150 mMNaCl and 1% of NP-40), homogenized, and sonicated for 30 min at 4°C to release associated MMPs. After sonication, proteins were precipitated with acetone by overnight incubation at −20°C. Extracts were then centrifuged at 14000 rpm for 10 min at 4°C and supernatants were collected. Protein precipitates were then diluted in PBS and the protein concentration of the samples was determined using a DC Protein Assay kit from Bio-Rad (500–0112). For each sample, 15 µg of protein was applied to non-reduced SDS-polyacrylamide gel electrophoresis using 10% gels containing 0.1% gelatin (bovine skin, Type B, SIGMA, G9391). The gels were electrophoresed under 80 V with a maximum intensity current of 120 mA, in a Mini-PROTEAN® Tetra Cell system from Bio-Rad. Following electrophoresis, the gel was washed twice with 2% v/v Triton X-100, to remove excess SDS, and incubated with MMP substrate buffer (50 mMTris–HCl, pH 7.5, 10 mM CaCl2) for 16 h. After incubation, the gel was washed with distilled water and stained with 0.1% w/v Coomassie Brilliant Blue solution (Sigma R-250). Areas of proteolysis appeared as clear bands against a blue background of gelatin substrate. Molecular mass determinations were made with reference to pre-stained protein standards. Stained gels were scanned and band densities were quantified by densitometric analysis (Quantity One Software, Bio-Rad). It should be noted that the gelatinolytic activity detected by this technique results from the presence of active MMPs, their inactive pro-enzymes and MMP-TIMP complexes (which are partly dissociated by SDS) [39].

#### Gelatinolytic activity in CDLs treated with galardin

To further assess the role of MMPs in CDL mutability, tissues in the different mechanical states were incubated with galardin. As there is considerable variability between individuals, we used two animals each for the uninhibited and galardin-inhibited conditions per gel. Per analysis, 10 CDLs (5 CDLs of each animal) were used for the uninhibited and inhibited conditions. More than five animals were used for each mechanical state. For the standard state, samples were maintained in a solution of 50 µM galardin in SW; stiff CDLs were kept in a solution of 50 µM galardin in AChSW; and compliant tissues were incubated with 50 µM galardin in PPSW. Control CDLs (from at least 5 animals per mechanical state) were left in SW, AChSW or PPSW alone. After overnight incubation at 4°C, tissues were prepared to measure their gelatinolytic activity as previously described in section 2.3.1. Overnight incubation with galardin was employed, because in an initial trial incubation for 10 min resulted in no discernible change in gelatinolytic activity. This appears to be at odds with the rapid effect of galardin on the mechanical properties of CDLs, as described below, but indicates that, to become enzymographically detectable, inhibition of MMP activity sufficient to affect CDL tensility may require amplification such as was afforded by the prolonged galardin treatment.

### Ethical Treatment of Animals

No specific permits were required for the described field studies since sea-urchins (*Paracentrotus lividus*) are invertebrates. This work was performed with a species that is not endangered or protected. The location of the field studies is also not privately owned or protected in any way.

### Statistical Analysis

All experiments were repeated at least five times. Statistical differences between CDLs in different functional states were determined using Kruskal-Wallis one-way analysis of variance with Dunn’s post-hoc test. All statistics were performed using GraphPad Prism 5 Demo software (version 5.02). Data are given as mean ± standard deviation (SD). Results were considered statistically significant when P<0.05.

## Results

### Mechanical Properties

#### Viscoelasticy of CDLs in different mechanical states

**Figure 2:**
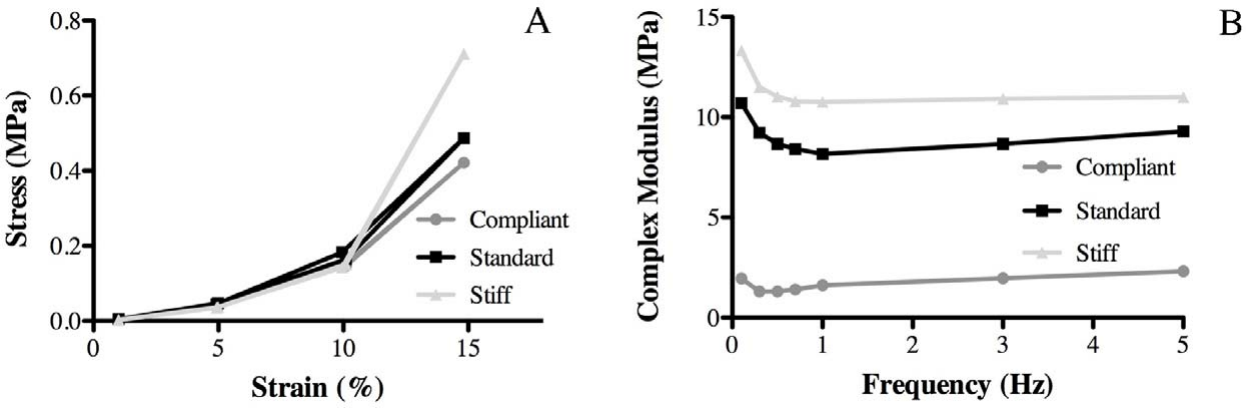
Mechanical properties of CDL. (A) Representative stress vs. strain curves of CDLs from one animal in the three mechanical states. (B) Effect of frequency on the complex modulus of CDLs from one animal in the three mechanical states tested at 13% strain.

Before evaluating the viscoelasticity of CDLs in the different mechanical states, the relationship between the maximum strain imposed on cyclically (constant frequency 1 Hz) loaded CDLs in different mechanical states and the resulting maximum stress was investigated (Fig.2A).

As is typical for collagenous tissues, the stress-strain curves were J-shaped, with a non-linear toe and heel region followed by a linear (constant stiffness) region, indicating that CDLs were more compliant at low strains and became stiffer as deformation progressed. This transition was abrupt and took place at 10% deformation. Fig. 2B shows the relationship between the cyclical loading frequency at a constant maximum strain of 13% and the complex modulus (E*) of CDLs in the three mechanical states. On the basis of these results, it was decided that a maximum strain of 13% and a loading frequency of 1 Hz should be employed in subsequent experiments.

**Figure 3:**
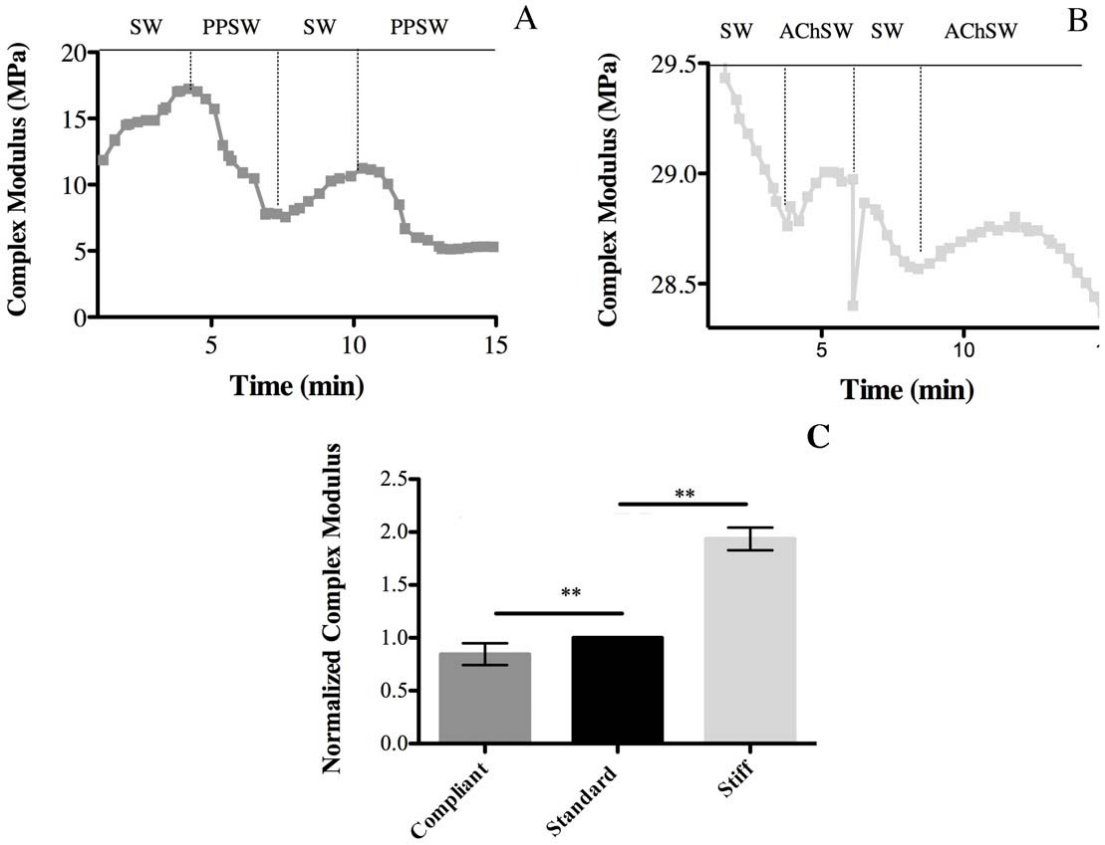
Effects of propylene phenoxetol (PPSW) and acetylcholine (AChSW) on the mechanical properties of the CDL. (A) Immediate effect of PPSW on a standard CDL. (B) Immediate effect of AChSW on a standard CDL. (C) Comparison of the normalized complex modulus of compliant (PPSW-treated), standard (untreated) and stiff (AChSW-treated) CDLs (n = 7).

In tests with CDLs in the standard state, the replacement of SW with PPSW resulted in a decrease in the complex modulus, which was partly reversible and repeatable (Fig. 3A, C, Table 1).

**Table 1:** Mean values ± S.D. of unnormalized complex modulus (n = 7).

Mean Complex Modulus before ACh stimulation (MPa)	Mean Complex Modulus after ACh stimulation (MPa)	Mean Complex Modulus before PPSW stimulation (MPa)	Mean Complex Modulus after PPSW stimulation (MPa)
12.6±12.23	20.34±9.17	22.8±13.8	16.74±12.31

In equivalent tests in which SW was replaced with AChSW, there was a reversible and repeatable increase in the complex modulus (Fig. 3B, C, Table 1).

In the present investigation, there was a high level of variation in the mechanical results generated by DMA, which is reminiscent of the inter-individual variability that has previously been reported in MCTs of *P. lividus* and other echinoderms [40], [41]. To compensate for this effect, which could have masked the influence of chemical agents on CDL mechanical behavior, for each mechanical condition (compliant, standard and stiff) we used five animals, and a minimum of three CDLs from each animal, one for each mechanical condition.

#### Effect of MMP inhibition on CDL viscoelasticity

**Figure 4:**
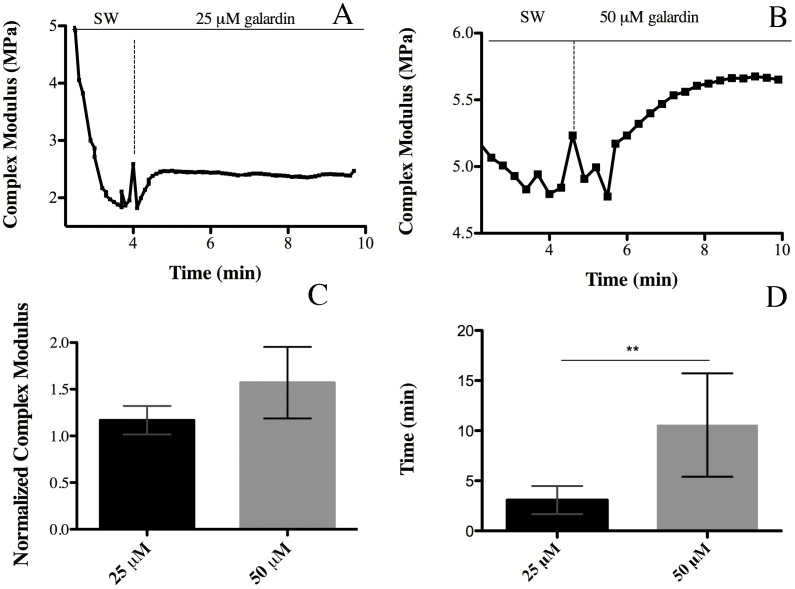
Effect of MMP inhibition. (A) Effect of 25 µM galardin on a standard CDL. (B) Effect of 50 µM galardin on a standard CDL. (C) Comparison of the effects of different galardin concentrations. Values were normalized against E* values obtained before galardin addition. (D) Comparison for different galardin concentrations of the time period between the addition of galardin and the return of E* to the pre-treatment value. The action of galardin was quantified by normalizing the maximum E* reached after galardin addition against the value just before the application of the chemical.

**Figure 5:**
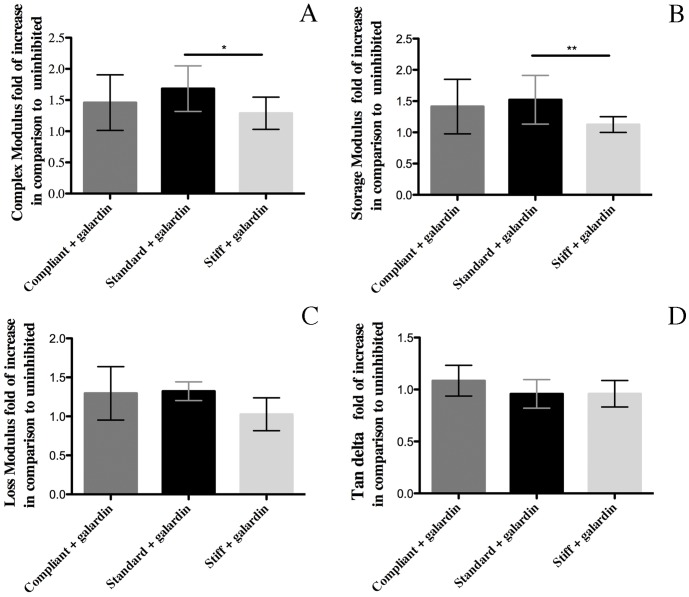
Effect of MMP inhibition on CDL viscoelasticity. (A) complex modulus, (B) storage modulus, (C) loss modulus and (D) tan delta of compliant, standard and stiff CDLs treated with 50 µM galardin in PPSW, SW, and AChSW respectively. The action of galardin was quantified by normalizing the maximum E* reached after galardin addition against the value just before the application of the chemical. The asterisk (*) represents statistically significant difference P<0.05 and the double asterisk (**) P<0.01.

When galardin was applied to standard CDLs, there was some instability due to addition of the solution, and then the stiffness (E*) increased quite rapidly to a maximum value, after which it decreased gradually, with E* returning, after up to 18 min, to roughly the value observed at the beginning of the test (Fig. 4A, B). The effect of MMP inhibition appeared to be concentration-dependent, the higher concentration of galardin (50 µM) producing both a higher (though not statistically significant) mean maximum E* and a longer mean response duration (which was statistically significant) in standard CDLs (Fig. 4A–D). Therefore the higher concentration was selected and used in further experiments with CDLs in the different mechanical states. As in standard CDLs, the MMP inhibitor enhanced rapidly the stiffness of compliant and stiff CDLs, which then decreased gradually to initial values (Fig. 5A).

The increase in E* caused by galardin was significantly greater in standard CDLs (mean fold of increase 1.57±0.38; N = 5) than in stiff CDLs (mean fold of increase 1.09±0.06; N = 5); the increase in E* of compliant CDLs (mean fold of increase1.5±0.47; N = 5) did not differ significantly from the other two (Fig. 5A). Increases in the storage modulus showed exactly the same pattern, while increases in the loss modulus and tan δ did not vary significantly between the three mechanical states (Fig. 5B–D). The increase in the E* of standard CDLs caused by galardin was not significantly different from that observed when standard CDLs were stimulated with AChSW (mean fold of increase 2.19±0.86; N = 5).

**Figure 6:**
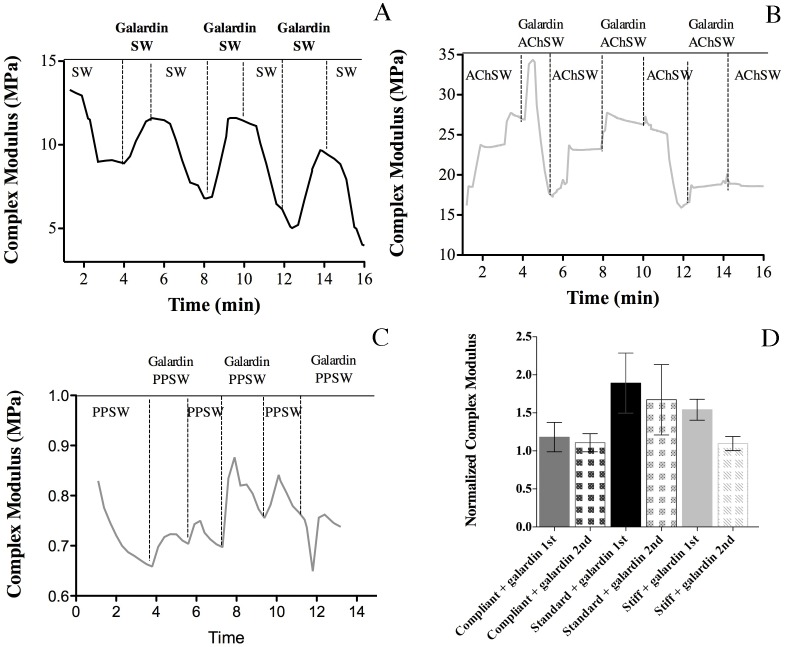
Reversibility of MMP inhibition. (A–C) Examples of recordings showing the reversibility of the galardin effect on (A) standard, (B) stiff and (C) compliant CDLs. (D) Normalized results. Tissues in compliant, standard and stiff states were stimulated with 50 µM galardin, washed and treated again with galardin. Data are expressed as means ± SD.

As the mutability phenomenon is reversible, further tests were performed in order to determine if the effect of the MMP inhibitor was reversible. The reversibility of the inhibitory effect of 50 µM galardin on standard, stiff and compliant CDLs was demonstrated by rinsing CDLs with sea water (SW), acetylcholine chloride in sea water (AChSW) or propylene phenoxetol in sea water (PPSW), respectively, after exposure to galardin (Fig. 6A–C). The inhibitory effect was reversible and could be subsequently repeated. Although in all three mechanical states the mean normalized stiffness of the second treatment was lower than that of the first, these differences were not significant (Fig. 6D).

### Gelatinolytic Activity of CDLs in Different Mechanical States and the Effect of Galardin

**Figure 7:**
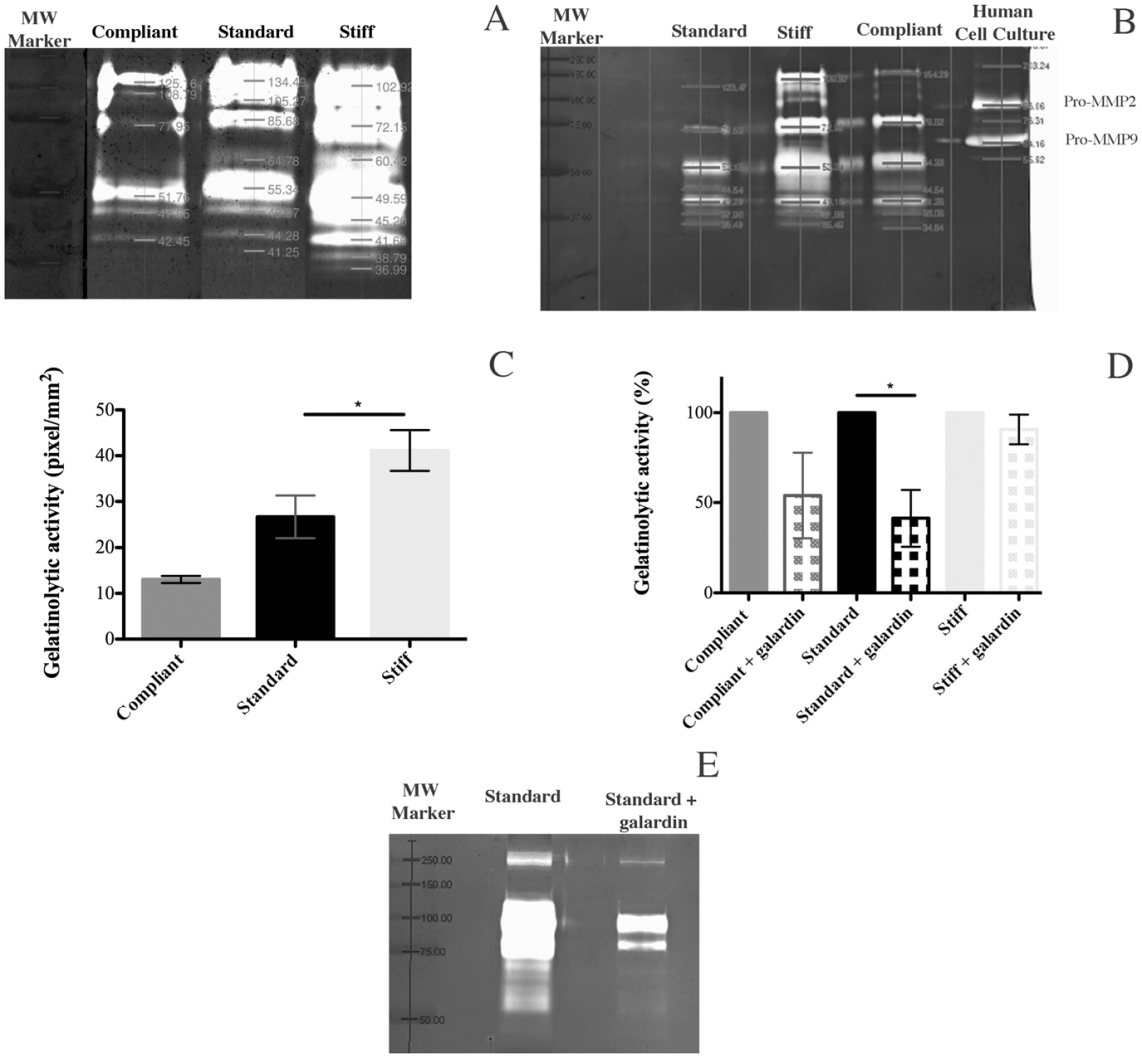
MMPs of CDLs in compliant, standard and stiff conditions visualized by gelatin zymography. (A) Zymogram showing pro-enzyme and active MMP band profile of CDLs in the three mechanical states. (B) Zymogram comparing the band profiles of CDLs, compared with human cell line showing MMP-2 and MMP-9 activity; this zymogram was included because of its very good band separation. (C) Optical density of MMP activity in CDLs in the three mechanical states. (D) Comparative densitometric analysis of scanned gels of CDLs in the different mechanical states with and without 50 µM galardin. Data are expressed as means ± SD. The asterisk (*) represents statistically significant difference P<0.05. MMPs were detected in more than six animals for each of the three mechanical states. (E) Zymogram comparing standard CDLs with and without galardin treatment.

A similar pattern of gelatinolytic bands with molecular weights ranging from 35 to 250 kDa was obtained for standard, compliant and stiff states (Fig. 7A, B, E). Stiff CDLs had clearly the highest level of gelatinolytic activity in all five zymograms (Fig. 7A, B), and densitometric analysis showed that there was a progressive increase in total gelatinolytic activity from the compliant to the stiff states (Fig. 7C). Where the resolution of the bands was high enough (Fig.7B), densitometric analysis indicated that the gelatinolytic activity of stiff CDLs was higher than that of standard and compliant CDLs at all molecular weights, the difference being particularly great at molecular weights above 53 kDa (up to six times higher in the example shown in Fig. 7B). This, however, could not be confirmed in all cases.

Overnight treatment of CDLs with 50 µM galardin had little effect on the mean normalized gelatinolytic activity of stiff CDLs, but caused a pronounced and statistically significant reduction in that activity of the standard CDLs, and caused a pronounced, although not statistically significant, reduction in that activity of the compliant CDLs (Fig.7 D, E).

## Discussion

### Basic Organization and Mechanical Properties of CDLs

The biomechanical behavior of the CDL is dependent on the composition and organization of its extracellular components. The CDL consists of parallel aggregates of collagen fibrils to which proteoglycans are attached, potentially serving as binding sites for molecules responsible for interfibrillar cohesion [2], [5], [19]. Available evidence suggests that interfibrillar cohesion is mediated by complexes of molecules, some constitutive and others regulatory [2], [19]–[21], [42]. The collagen fibril bundles (‘fibers’) provide the ECM with mechanical integrity and strength, and are delimited by a network of fibrillin-containing microfibrils, which is an elastic component that may provide resilience and help the CDL to re-shorten after elongation [2], [5], [11], [12]. As for other mutable collagenous structures, the mechanical adaptability of the CDL depends on the modulation of interfibrillar cohesion and not on changes in the organization or mechanical properties of the collagen fibrils [5], [36]. There is evidence that the juxtaligamental cells (JLCs) control this process in the CDLs and other MCTs [2], [36].

### Effect of MMP Inhibition on CDL Viscoelasticity

During the development, growth and remodelling of load-bearing connective tissues in mammals, fibrillar collagens are secreted and continuously degraded by MMPs [23]–[25]. It is notable that the sea urchin genome includes at least 26 MMP genes with significant similarity to those of mammals [43], [44]. If MMPs have a role in the variable tensility of MCTs, they are likely to contribute to MCT *destiffening* by hydrolyzing components of the interfibrillar crosslink complexes and therefore their inhibition should reverse and/or block destiffening.

The MMP inhibitor used in this investigation was galardin, or GM6001, which is highly potent against mammalian MMP-1, -2, -3, -8 and -9. It has a collagen-like backbone, to facilitate binding to the active site of MMPs, and a hydroxamate structure, which chelates the zinc ion located in the catalytic domain of MMPs [45]–[50]. Such chelation results in alteration of the attached MMP molecular conformation, blocking its proteolytic activity against extracellular matrix components and other substrates [45]–[50]. The involvement of MMPs in CDL mutability was supported by our finding that galardin (50 µM) increased the stiffness (E*) and storage modulus (É) of CDLs in all three mechanical states, although it had a significantly lower effect on stiff CDLs. The preferential enhancement of the storage modulus and lack of effect on the loss modulus indicates that externally applied force was transferred more efficiently to the stiff and inextensible collagen fibrils, which would be a consequence of the strengthening of interfibrillar cohesion. The involvement of MMPs in CDL mutability was also suggested by the observations that (1) the increase in E* of standard CDLs following galardin treatment was not significantly different from that observed when standard CDLs were treated with AChSW and (2) that, as is the case for *in vivo* changes in mechanical properties, the action of the MMP inhibitor was reversible.

In response to galardin, CDL stiffness increased to a maximum and then returned to pre-treatment values after up to 18 min (though the duration of the responses was very variable). The reason for the transient nature of galardin’s effect is unclear. As available evidence indicates that galardin retains its MMP-inhibitory action in experimental solutions for at least days [48], it is likely that the transiency is due to the CDLs themselves. The progressive reduction in stiffness shown by some CDLs before any agent was added (see e.g. Fig. 6 B, C) suggests that the protracted oscillatory stress to which the CDLs were subjected could by itself cause interfibrillar slippage, and it is possible that this overwhelmed the galardin-induced increase in crosslink density after variable periods of time. It may also be relevant that the galardin concentrations employed in the experiment were very low, 50 µM being “therapeutically attainable” [48]. It is also possible that the galardin-induced responses, their transiency and their variability result partly from the inhibition of MMP effects on cellular rather than extracellular components of the CDLs. There is, for example, evidence that certain MMPs can inactivate calcium channels in mammalian vascular smooth muscle [49]. Specific experimentation would be required, however, to elucidate any involvement of such a mechanism in the regulation of CDL tensility.

**Figure 8:**
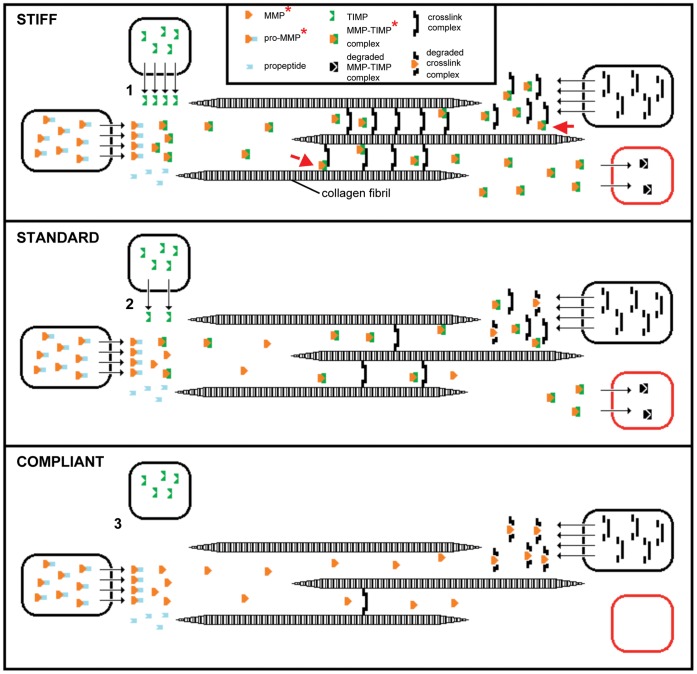
Hypothetical model of the involvement of MMPs in MCT mutability. It is known that MCTs consist of discontinuous collagen fibrils crosslinked by complexes of molecular components, and that changes in the mechanical properties of MCTs result from rapid changes in the strength of the interfibrillar cohesion that is mediated by these crosslink complexes. We found that the synthetic MMP inhibitor, galardin, increased the stiffness of CDLs in all three mechanical states, which suggests that in all three states there is ongoing MMP activity that has the potential to degrade components already incorporated into existing crosslink complexes and components that have been secreted but not yet incorporated, and ongoing synthesis and release of new crosslink components. The model acknowledges that MMPs are synthesized and secreted as inactive pro-enzymes, then activated extracellularly by proteolytic removal of the pro-peptide domain [39], [51], [52]. It is envisaged that crosslink components are synthesized and secreted separately, then assembled extracellularly to form functional complexes. The black boxes represent cells, although it should be noted that the three processes do not necessarily occur in different cell-types. The red box represents the process by which MMP-TIMP complexes are removed and degraded. For the sake of simplicity, the model assumes that activated MMPs and new crosslink components reach the extracellular environment at a constant rate. It is hypothesized that interfibrillar cohesion is regulated only through changes in the rate at which an endogenous MMP inhibitor (which we assume is a TIMP-like molecule) is released into the extracellular environment. In the stiff state there are high levels of TIMP secretion (1), MMP inhibition and crosslinking. In the standard state there are intermediate levels of TIMP secretion (2), MMP inhibition and crosslinking. In the compliant state there are low levels of TIMP secretion (3), MMP inhibition and crosslinking. Also represented is the possibility that an endogenous inhibitor could function as a component of the crosslink complex (red arrows) and thus have a dual function (which may apply to TIMP-like tensilin). The model also assumes that the production of MMP-TIMP complexes exceeds the rate of removal and degradation of MMP-TIMP complexes, which would account for the positive correlation between degree of CDL stiffness and total gelatinolytic activity. The components marked with a red asterisk contribute to the gelatinolytic activity of CDLs as quantified by gelatin zymography.

If MMPs contribute to the mutability of the CDL and of MCTs in general, their primary role could be reactive or constitutive: either it is only when the tissue is destiffening that activated MMPs are present in the extracellular environment and degrade their substrates, or activated MMPs are continuously present in the extracellular environment, and what varies is the extent to which the enzymes are inhibited, less inhibition resulting in destiffening and more inhibition resulting in stiffening. We found that galardin stiffened CDLs in all three mechanical states, implying that in all three states there is (1) ongoing MMP activity, which thus supports the constitutive model, and (2) ongoing production of crosslink components. The significantly weaker effect of galardin on stiff CDLs than on standard or compliant CDLs would be expected, since in stiff CDLs MMP activity would already be greatly suppressed, together with the obvious fact that stiff CDLs must have much less capacity to stiffen further than standard and compliant CDLs. The constitutive model is illustrated in Fig. 8 (which is another “three-state” model: see Motokawa and Tsuchi, 2003) [51]. We hypothesize that the stiffness of the CDL is adjusted through the modulation of constitutive MMP activity. Since in the collagenous tissues of other animals, activated MMPs are constrained mainly by enzyme inhibitors, such as TIMPs [39], [52], [53] we further hypothesize that the control of CDL stiffness depends ultimately on the rate of release into the extracellular environment of endogenous MMP-inhibitors.

One aspect of our results that still requires explanation is the insignificant difference between the effects of galardin on CDLs in the compliant and standard states. At the moment we can only speculate that this relates to an asymmetry in the changes in MMP activity responsible for the compliant to standard and standard to stiff shifts, i.e. the increase in crosslink density associated with the compliant to standard shift, and the corresponding degree of MMP inhibition, may be much less (and perhaps too low to be detected by enzyme zymography) than the increase in crosslink density and degree of MMP inhibition that bring about the standard to stiff shift. It is also possible that the compliant to standard shift relies mainly on a mechanism other than MMP inhibition. It appears that different mechanisms are responsible for the compliant to standard and standard to stiff changes occurring in holothurian dermis [21].

### Gelatinolytic Activity of CDLs in Different Mechanical States and the Effect of Galardin

MMPs have a wide spectrum of activities: in particular, they degrade all extracellular matrix components, as well as growth factors, pro- and anti-inflammatory cytokines and chemokines, and they also modify apoptotic signals and regulate the immune response. As a consequence, they play a fundamental role in tissue morphogenesis, wound healing, tissue repair and remodeling, and in the progression of some diseases such as cancer [22]–[25], [31].

Included amongst the potential substrates of some MMPs is gelatin, i.e. collagen that has been denatured in the first stage of the degradation process [24], [39], [52]. We analyzed the gelatinolytic activity and distribution pattern of CDLs in different mechanical conditions using gelatin-zymography. This detects the presence of activated MMPs, inactive pro-MMPs and some MMPs previously bound to TIMPs [39]. No qualitative differences in the general pattern of gelatinolytic activity were observed between standard, stiff and compliant conditions. There were, however, significant quantitative differences, with a progressive increase in total gelatinolytic activity in the compliant, standard and stiff states (Fig. 7A–C). The absence of a *P. lividus* protein database and differences between the proteolytic patterns of the CDL and mammalian tissues prevented identification of the distinct enzymatic bands, although comparison with the gel of a human cell line suggested that mammalian MMP-2-like and MMP-9-like enzymes were not present.

It appeared that the higher gelatinolytic activity of stiff CDLs was particularly prominent at higher molecular weights, which probably resulted from the presence of MMP-MMP complexes and MMPs bound to endogenous inhibitors [39].The presence of MMP-MMP and MMP-TIMP complexes is associated with the inactivation and removal of MMPs, the latter occurring at least partly by endocytosis [39], [52]. This positive correlation between degree of stiffness (compliant<standard<stiff) and increasing levels of higher molecular weight complexes (resulting from increasing inhibition of activated MMPs) is consistent with our model (Fig. 8). More difficult to reconcile with this model is the apparent positive correlation between degree of stiffness and the total gelatinolytic activity (Fig. 7C), which seems to be the opposite of what would be expected if MMPs cause destiffening. However, it has to be stressed again that gelatinolytic activity results from the presence of not only *active* MMPs, but also of their *inactive* pro-enzymes and the molecular complexes associated with the removal and degradation of MMPs (i.e. MMP-TIMP and MMP-MMP complexes) [39]. It is possible that in the standard and stiff states, in which there is increasing inhibition of MMPs, there is a progressive accumulation of MMP-TIMP and MMP-MMP complexes in the extracellular environment, perhaps because the rate of production of these “disposal complexes” exceeds their rate of removal and degradation (Fig. 8).

The effects of galardin on the gelatinolytic activity of the CDLs provide further support for the model illustrated in Fig. 8. Galardin did not affect the gelatinolytic activity of stiff CDLs, which is expected, since, according to our model, MMP activity of stiff CDLs would already be maximally inhibited. Our model predicts that in both the standard and compliant states there should be significant MMP activity, and in accordance with this we found that galardin significantly reduced the total gelatinolytic activity of standard CDLs, although its effect on compliant CDLs was marked but not statistically significant.

As discussed in connection with the similar pattern of effects of galardin on the mechanical properties of the CDL, the lack of differentiation between the gelatinolytic activities of compliant and standard CDLs after galardin treatment may be due to an asymmetry in the changes in crosslink density and MMP inhibition associated with the compliant to standard and standard to stiff shifts, or because MMP inhibition is not involved in the compliant to standard shift.

### Identity of the Endogenous Inhibitor

According to our hypothesis, the stiffness of the CDL and other echinoderm MCTs is determined by the rate of secretion of one or more endogenous MMP inhibitors. It may of course be relevant that tensilin – a possible regulatory protein from MCT that has been fully sequenced – shows homologies to mammalian TIMPs. Holothurian tensilin aggregates collagen fibrils and stiffens samples of whole dermis. It is stored intracellularly and has been immunolocalized to the intracellular granules of juxtaligamental-like cells in holothurian dermis [19]. Whilst it is the role of tensilin as a potential regulatory stiffening agent that has been emphasized, it is notable that it cannot induce maximal stiffening (equivalent to the standard to stiff shift) of holothurian dermis, though another incompletely characterized protein can do this [20], [21]. Furthermore, the homology of tensilin’s deduced peptide sequence to mammalian TIMPs is stronger in its N-terminal domain [19], which in mammalian TIMPs forms the unit that inhibits MMPs [51], [52]. It is therefore possible that tensilin retains the capacity to inhibit MMPs and even that this is its main function. Like mammalian TIMP-3, tensilin binds to the sulphated GAGs of ECM components through its C-terminal domain [19], which suggests that tensilin may be a TIMP that has evolved the additional capacity to act as an adjuvant stiffener. We therefore suggest in our model (Fig. 8) that the endogenous regulatory inhibitors are TIMPs that may also contribute to crosslink complexes.

### Comparison with the Uterine Cervix

The mammalian uterine cervix can be regarded as another example of a mutable collagenous structure that demonstrates reversible changes in stiffness. Its mutability differs from that of echinoderm MCTs in its longer time course, among other aspects. It occurs in three stages: cervical ‘ripening’ begins about four weeks before birth and results in expansion of the cervical canal to 3–4 cm, followed by cervical dilatation to 10 cm during parturition itself, which occurs within hours, and then by the recovery of cervical stiffness, which takes days. These changes in mechanical properties result from modification of the biochemical composition and structure of the cervix and by MMP-dependent degradation of collagen fibrils and other ECM components. The MMP activity is modulated by endogenous inhibitors, including TIMPs, which become particularly important as a brake on the degradation process at the end of parturition [31], [34], [54]. The involvement of MMPs in cervical destiffening is reactive: the increase in MMP activity results from a dramatic rise in MMP concentrations following increased synthesis by neutrophilic leukocytes, and is under mainly endocrine control [31], [54]. Thus regulation of MMPs at the transcriptional level is an important component of the cervical mutability phenomenon. In contrast to this, we postulate that in echinoderm MCTs the regulation of MMP activity occurs mainly at the extracellular level and is determined by the rate of inhibitor secretion. This provides a much faster responsiveness (within timescales of<1 s to minutes) than could a mechanism dependent on the adjustment of protein synthesis and is amenable to nervous control, which allows changes in the mechanical state of MCTs to be coordinated with the activity of contractile systems.

We believe that Fig. 8 illustrates the simplest model that can integrate our results with information derived from other MCTs. Aspects of it are admittedly speculative, but these are testable by further experimentation. The uniqueness of echinoderm MCT cannot be exaggerated: collagenous connective tissue that is directly innervated by the motor nervous system, and that can alter drastically and reversibly its mechanical properties within short physiological timescales, appears to have evolved only in the phylum Echinodermata (though a “pre-neural” version of the phenomenon occurs in the Porifera [55]). Despite this restricted taxonomic distribution, the investigation of MCT has the potential to provide information of widespread biomedical applicability [2], [56].
